# Simultaneous Application of Proximal Fibular Osteotomy and Unicondylar Knee Arthroplasty

**DOI:** 10.7759/cureus.4763

**Published:** 2019-05-28

**Authors:** Nihat Demirhan Demirkıran

**Affiliations:** 1 Orthopaedics, Kütahya Health Sciences University School of Medicine, Kütahya, TUR

**Keywords:** medial compartment arthritis, proximal fibular osteotomy, unicondylar knee arthroplasty

## Abstract

Proximal fibular osteotomy (PFO) is a simple, easy and cheap procedure consisting of removing a 10 mm piece of fibula 6 to 9 cm below the fibular head. Proximal osteotomy of the fibula weakens the lateral fibular support and leads to a correction of the varus deformity and provides a widening on the medial joint space. Unicondylar knee arthroplasty (UKA) is intended to treat isolated medial compartment arthritis. However, knees with a varus deformity and tight medial joint space might cause both technical difficulties and poorer outcomes. A 68-year-old female with complaints of pain on the medial side of both knees for more than two years underwent bilateral UKA. While inserting the trial meniscal bearing was not easy due to the varus tight knee, at that stage, instead of performing a deeper cut, a PFO procedure was considered which provided widening of the medial joint space. We report, to our knowledge, the first simultaneous application of these two procedures. Despite being a single case with very short term results, our results suggest that the combination of PFO and UKA may reduce the loads over the implants on the medial compartment based on the widening of the joint space and varus deformity correction.

## Introduction

Osteoarthritis (OA) is a chronic, progressive degenerative disease of synovial joints that causes progressive loss of articular cartilage causing pain and joint deformity. OA of the knee is one of the most common orthopedic problems of the elderly population with a high incidence of 30% after the age of 60. It is the most common musculoskeletal complaint worldwide and is associated with significant health and welfare costs [1]. As life expectancy continues to increase, the ratio of the older population also rises, leading to a higher arthritis prevalence. It is estimated that OA will become the fourth leading cause of disability in the near future [2-3]. Various factors have been accused in etiology including articular trauma, muscle weakness, obesity or metabolic syndrome, increased age, genetics, and race. Patients usually present with function-limiting knee pain, stiffness, crepitation, and decreased range of motion. Although patient history, physical examination, and laboratory studies are also included in diagnostic criteria, plain radiographs with typical findings such as joint space narrowing, osteophytes, eburnation of bone, and subchondral sclerosis provide sufficient data for OA diagnosis in most cases [4]. Symptomatic knee OA is more common in the medial compartment. Anatomical and mechanical factors are both thought to be associated with this higher incidence [5-6]. Normal alignment of the lower limb directs weight bearing forces to the medial compartment of the knee and the medial compartment bears 60% to 80% of the load [7]. Besides the relatively thinner cartilage and narrower meniscal surface area makes the medial compartment more vulnerable to arthritic changes. Consequently, medial compartment arthritis is the usual starting point for knee OA.

High tibial osteotomy (HTO) and unicondylar knee arthroplasty (UKA) are common surgical procedures for the treatment of knee OA with satisfying results. However, both of these surgeries are technically demanding procedures, having risk of complications, and are dependent on implants with significant costs. Proximal fibular osteotomy (PFO) is a simple, easy and cheap procedure consisting of removing a 10 mm piece of fibula 6 to 9 cm below the fibular head. It was described incidentally in the early 2000s, based on follow-up observations of prisoners with medial arthritis of the knee who had relief in symptoms, after proximal fibular fractures frequently encountered in riots [8]. The main idea behind the procedure was that the medial part of the knee which tends to collapse with increasing age has only one single cortex support whereas the lateral side of the knee is supported by three cortices, one of tibia and two of fibula [9]. Proximal osteotomy of the fibula weakens the lateral fibular support and leads to a correction of the varus deformity. In the following case, we report the simultaneous application of PFO and UKA. To our knowledge, this is the first case to combine these two surgical procedures in order to treat medial OA of the knee. The patient provided written informed consent for print and electronic publication of this case report.

## Case presentation

**Figure 1:**
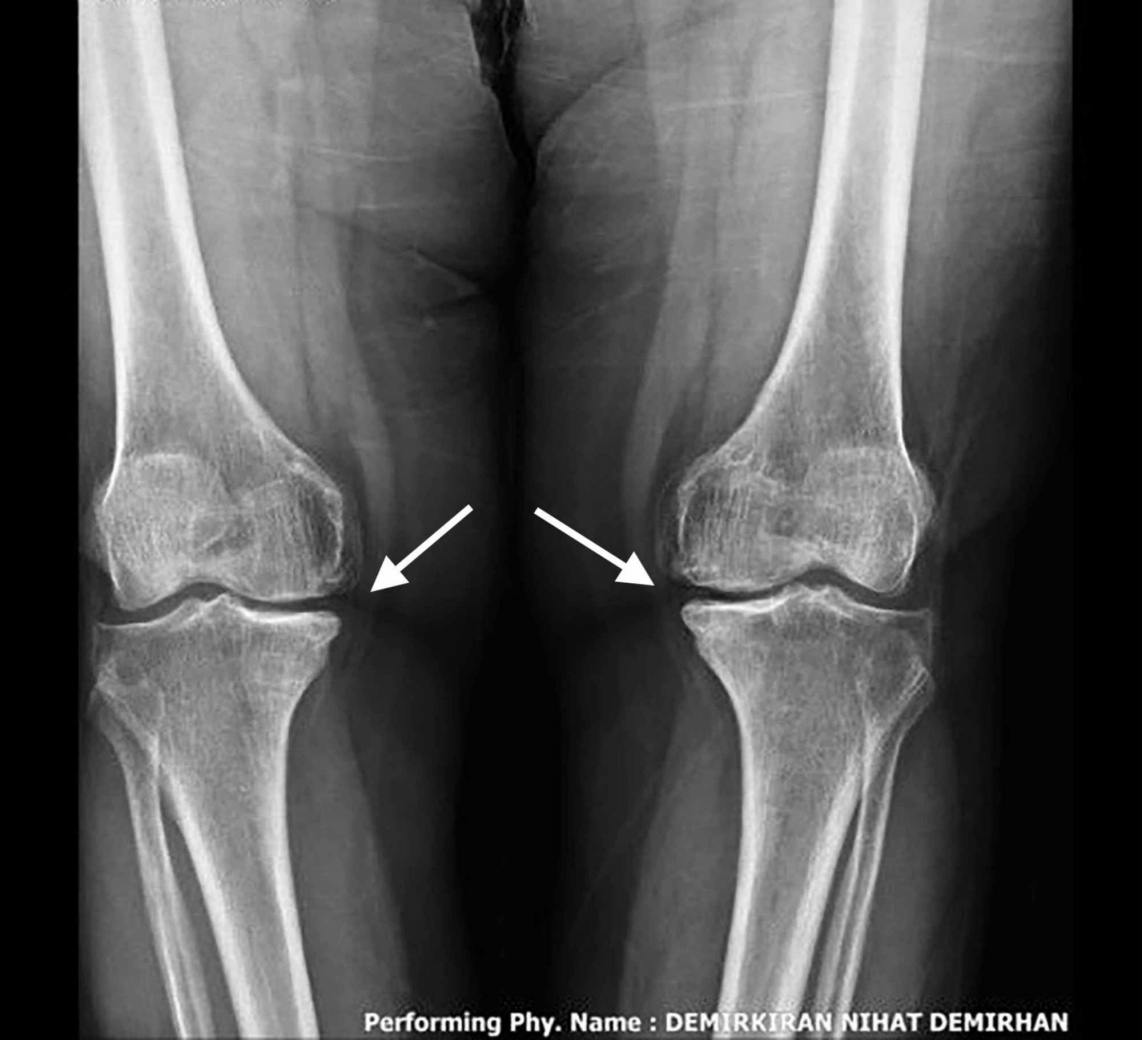
Preoperative antero-posterior (AP) radiograph of both knees; arrows indicate the medial joint space narrowing and osteophyte formation

A 68-year-old female presented to the orthopedics department with a history of pain on the medial side of both knees for more than two years. Physical therapy, rehabilitation, and education programs along with lifestyle changes were given to the patient; however, none were effective on her complaints. She had tenderness over the medial side of both knees with decreased range of motion. Preoperative visual analog scale (VAS) and American Knee Society (AKS) scores were similar; 8 and 7 VAS scores; 77 and 80 AKS scores for right and left knees, respectively. No symptoms regarding the lateral or patellofemoral compartment were noted. Physical examination revealed aggravated pain with palpation of the medial joint space. Radiographs demonstrated isolated medial arthrosis of the knee (Figure 1). 

**Figure 2:**
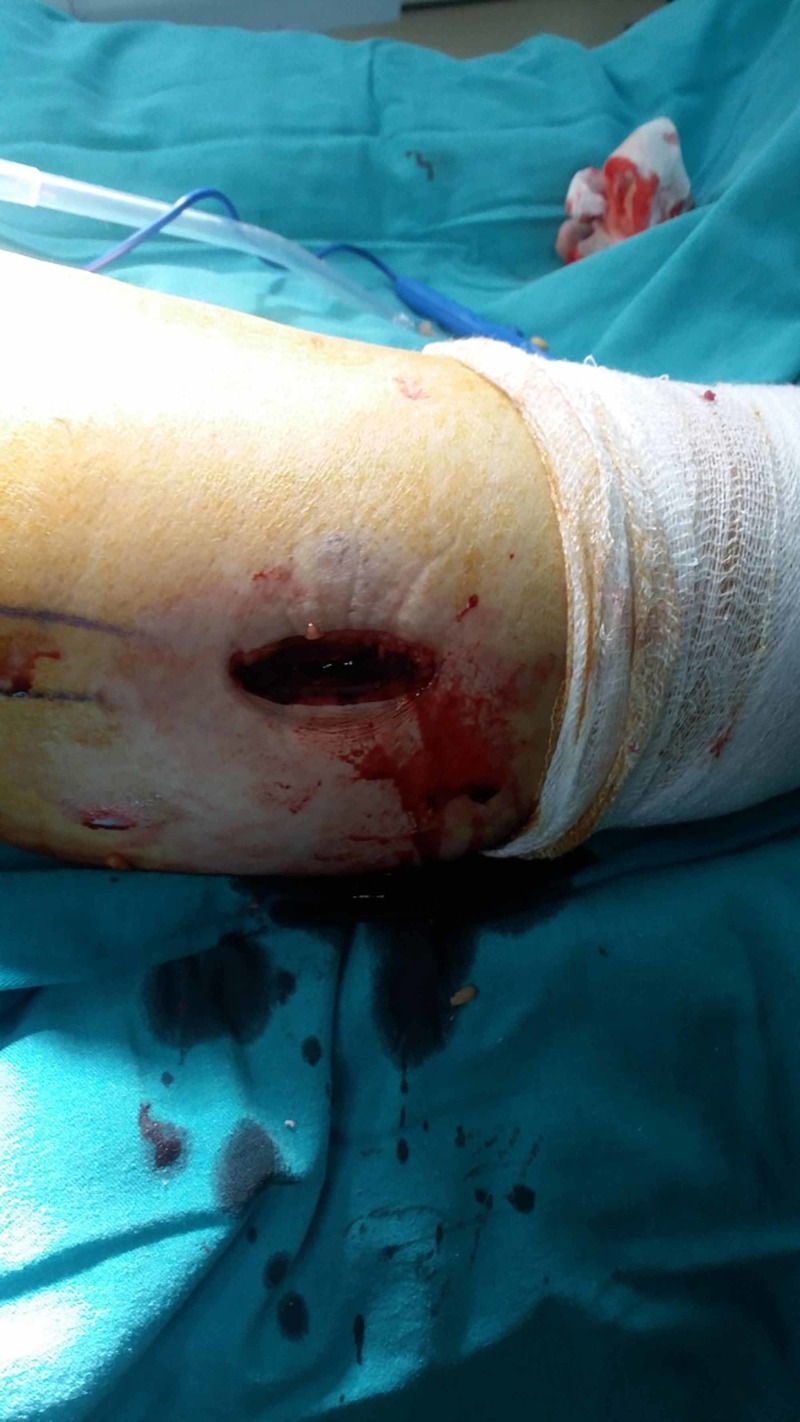
Intraoperative view of fibular incision 10 cm distal to the fibular head

The patient was elected for bilateral UKA surgery. Starting from the right knee, a medial parapatellar approach was used and after osteophyte excision, tibial plateau resection was performed with the guidance of femoral sizing spoon (Oxford Partial Knee Phase III, Biomet Zimmer Ltd., Bridgend, UK). Using a 12-mm wide oscillating saw blade, the plateau, showing typical anteromedial osteoarthritis, was excised. After femoral cuts and appropriate millings trial, tibial and femoral components were applied. However, inserting the trial meniscal bearing was not as smooth and easy as we were used to, possibly due to the varus tight knee. At that stage, instead of performing a deeper cut, we considered a PFO. A 5-cm skin incision was made targeting the fibular segment 10 cm distal to the fibular head (Figure 2).

**Figure 3:**
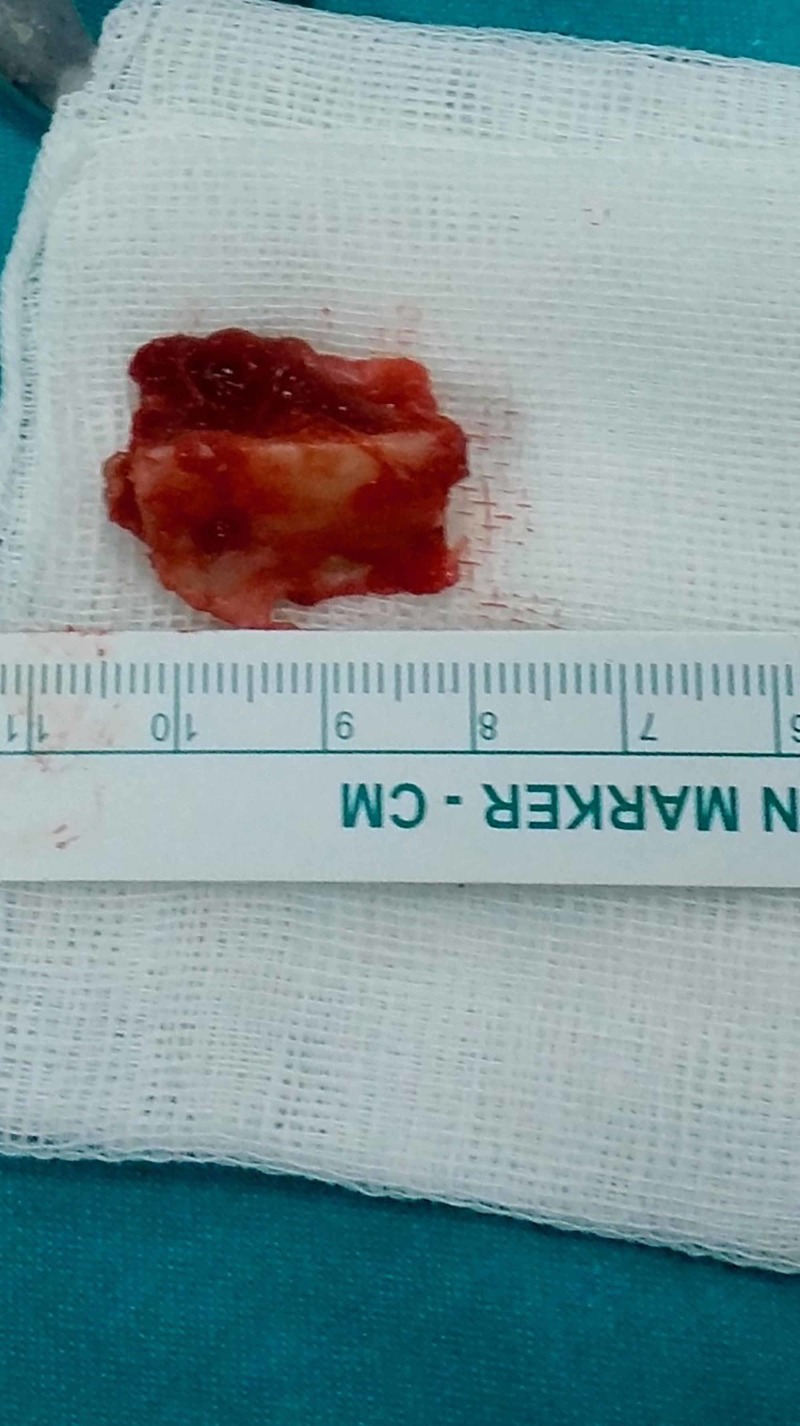
Resected fibular bone segment of 2 cm

Dissecting between peroneus and soleus muscles fibula was reached and a 2-cm bone was excised using the narrow oscillating saw blade (Figure 3).

**Figure 4:**
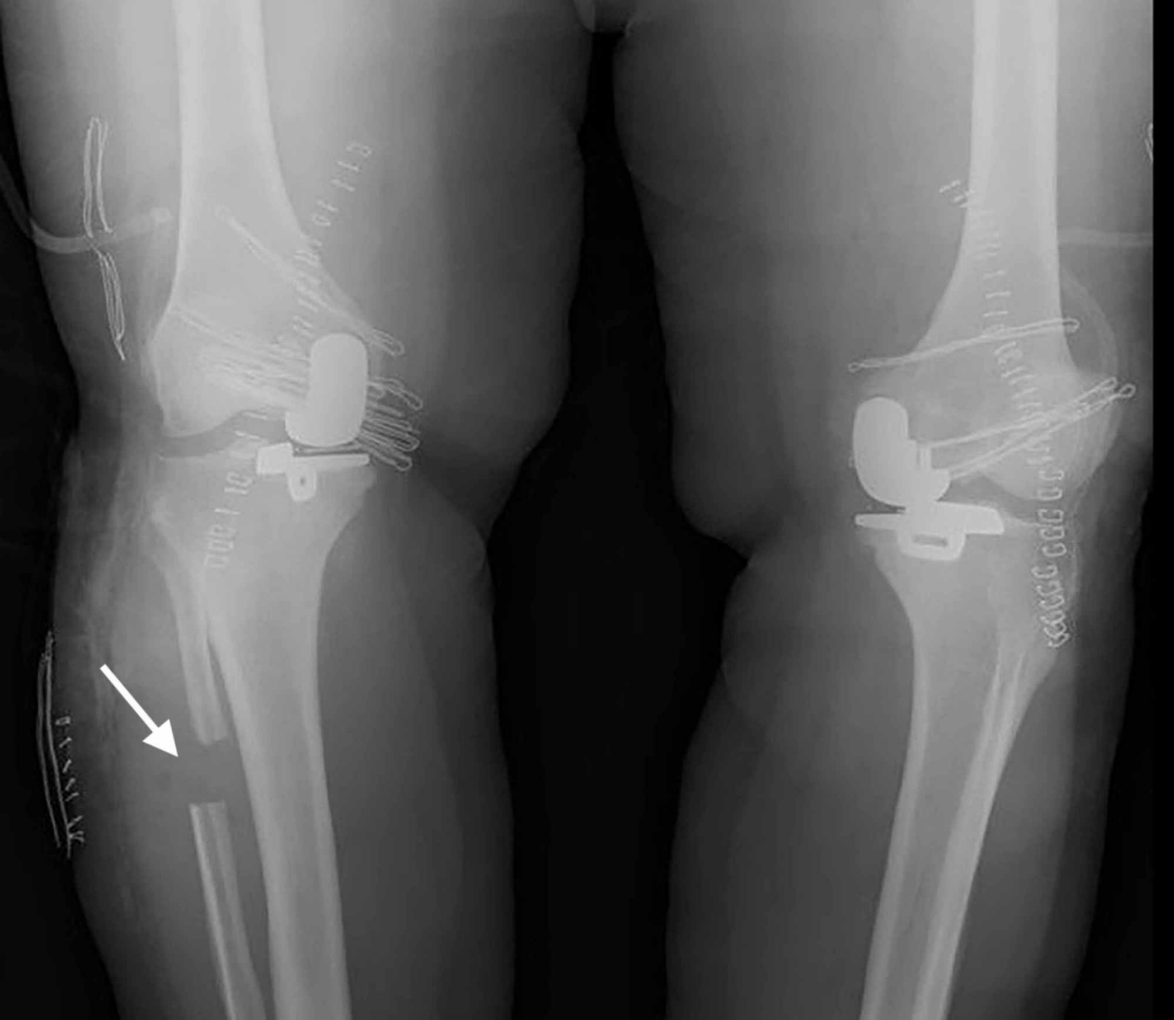
Postoperative antero-posterior (AP) radiograph of both knees; arrow indicates the proximal fibular osteotomy site. Note the staples of the short incision on lateral aspect

Under image intensification, the knee was forced on valgus and the widening of medial joint space was observed. With the same size trial components, another attempt to insert the meniscal bearing was found successful. With the bearing in place, manipulation of the knee through a full range of motion demonstrated the stability of the joint, security of the bearing, and absence of impingement. Final components were applied and the wound was closed in a routine manner. Subsequently, the left knee was operated with the same surgical technique; however, the meniscal bearing could be inserted without the need for a PFO (Figure 4).

**Figure 5:**
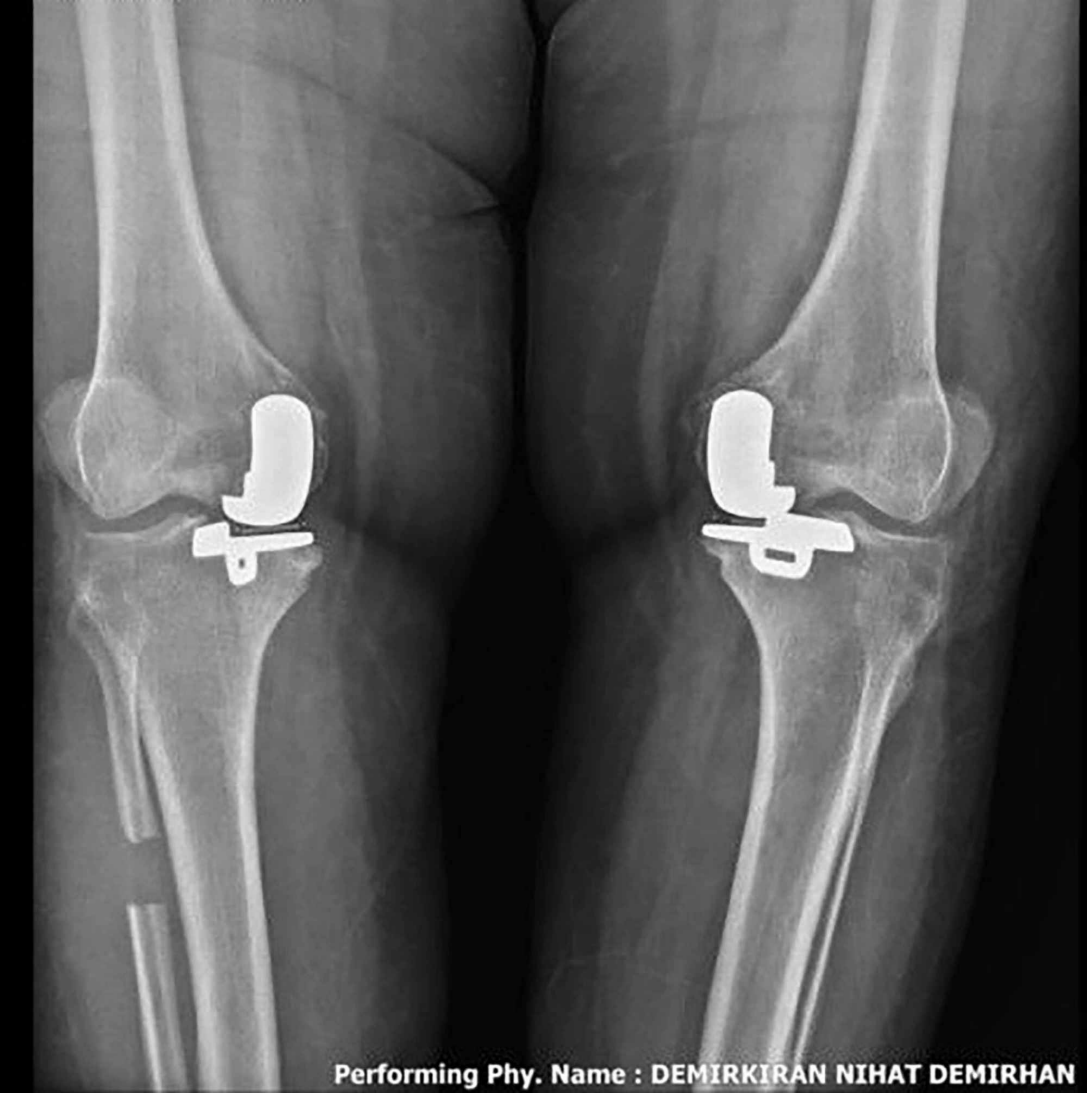
Full weight-bearing antero-posterior (AP) radiograph of both knees at six week follow-up

The patient was walking full weight bearing on postoperative day one and sutures were removed at the third-week follow-up (Figure 5).

At six weeks follow-up, she had slightly better VAS and AKS scores for that osteotomized right side than her left knee. VAS scores improved from 8 to 2 for the right side compared to 7 to 3 for the left side. AKS scores also increased for right and left knees to 151 and 144, respectively (Table 1). 

**Table 1:** Preoperative and postoperative radiographic, pain and functional data of our patient FTA: Femorotibial angle, VAS: Visual analog scale, AKSS: American Knee Society Score (knee score + functional score), Preop: Preoperatively, Postop: Postoperatively.

	Preop	Preop	Postop	Postop
	R	L	R	L
FTA	185	184	178	181
VAS	8	7	2	3
AKSS	37+40	40+40	81+70	74+70

## Discussion

UKA is a very effective procedure for medial compartment arthrosis. It is proven to improve functions and relieve knee complaints. However, it is also an expensive, technically demanding, and complex major surgery. Besides secondary revision to total knee arthroplasties may be necessary. PFO is a relatively simple surgical procedure with advantages such as short operating time and easy surgical technique. Moreover, it is an economical procedure without the necessity of any expensive implants.

The first study regarding PFO in the literature by Yang et al. demonstrated improved radiographic and functional results of 110 patients after more than two years follow-up [10]. The authors attributed this satisfying results to the shift of the loading forces from medial to the lateral compartment as a result of elimination lateral fibular support and correction of varus deformity. They suggested that the nonuniform settlement of the tibial plateau, which is caused by lateral support of the fibula, is the main factor responsible for mechanical axis shift and subsequent degenerative changes and fibular osteotomy and resection would solve this nonuniform settlement and varus deformity caused by this lateral support.

Wang et al. also found improved functional scores and pain relieve on 47 patients after PFO [11]. They also observed an increase in medial joint space and correction of lower extremity alignment in radiographic measurements. They tried to explain these successful outcomes with the redistribution of the load after removal of the fibula which carries one-sixth of the body weight. The results of this study showed a significant widening in the medial knee joint space postoperatively. They also associated these favorable outcomes with the correction of varus deformity as a result of lateral fibular support removal. In our case, the narrow joint space on the medial side presented as a difficulty on a varus aligned knee. The widening of the medial compartment as published in the literature was the main idea that made us consider a PFO to implant the meniscal bearing insert more easily. Under fluoroscopy guidance, we could observe the medial joint space widening after the osteotomy, and also prove it with the feeler gauge measurements. Along with the intraoperative deformity correction and joint space opening, we observed a permanent change in varus alignment. Although preoperatively both lower extremities had similar varus deformity angles, femorotibial angle for the osteotomized right knee was a little bit lower than the left side. In addition, the right knee had slightly better pain and functional outcomes in terms of VAS and AKS scores, as seen in Table 1.

## Conclusions

Despite the very short follow-up duration, this single case allowed us to compare the results of simultaneous PFO and UKA application with UKA alone on each knee of the same patient. Addition of this very simple and short procedure did not cause any adverse events including haematoma, nerve injury, or infection; neither did it cause dislocation of the meniscal bearing insert. The potential benefits of the combination of PFO and UKA could be the reduction of loads over the implants on the medial compartment based on the widening of the joint space and varus deformity correction. Although larger studies with longer follow-up times are needed, our case demonstrated promising short term results and a very simple solution to insert the meniscal bearing implant without applying too much pressure.

## References

[1] Hinman RS, Hunt MA, Creaby MW, Wrigley TV, McManus FJ, Bennell KL (2010). Hip muscle weakness in individuals with medial knee osteoarthritis. Arthritis Care Res.

[2] Felson DT, Naimark A, Anderson J, Kazis L, Castelli W, Meenan RF (1987). The prevalence of knee osteoarthritis in the elderly. The Framingham Osteoarthritis Study. Arthritis Rheum.

[3] (2019). The world health report 2002: reducing risks, promoting healthy life. World Health Organization.

[4] Altman R, Asch E, Bloch D (1986). Development of criteria for the classification and reporting of osteoarthritis: classification of osteoarthritis of the knee. Arthritis Rheum.

[5] Dearborn J, Eakin C, Skinner H (1996). Medial compartment arthrosis of the knee. Am J Orthop.

[6] Bartel DL (1992). Unicompartmental arthritis: biomechanics and treatment alternatives. Instr Course Lect.

[7] Wu L, Hahne HJ, Hassenpflug T (2004). A long-term follow-up study of high tibial osteotomy for medial compartment osteoarthrosis. Chin J Traumatol.

[8] Prakash L (2019). PFO - Proximal Fibular Osteotomy in Medial Compartment Arthritis of the Knee with Varus Deformity.

[9] Zhang Y, Li C, Li J (2014). The pathogenesis research of non-uniform settlement of the tibial plateau in knee degeneration and varus. J Hebei Med Univ.

[10] Yang ZY, Chen W, Li CX (2015). Medial compartment decompression by fibular osteotomy to treat medial compartment knee osteoarthritis: a pilot study. Orthopedics.

[11] Wang X, Wei L, Lv Z (2017). Proximal fibular osteotomy: a new surgery for pain relief and improvement of joint function in patients with knee osteoarthritis. J Int Med Res.

